# Unmet need for family planning and associated factors among adolescent girls and young women in Ethiopia: a multilevel analysis of Ethiopian Demographic and Health Survey

**DOI:** 10.1186/s40834-022-00211-x

**Published:** 2023-02-06

**Authors:** Desale Bihonegn Asmamaw, Wubshet Debebe Negash

**Affiliations:** 1 Department of Reproductive Health, Institute of Public Health, College of Medicine and Health Sciences, University of Gondar, P.O.Box: 196, Gondar, Ethiopia; 2 Department of Health Systems and Policy, Institute of Public Health, College of Medicine and Health Sciences, University of Gondar, Gondar, Ethiopia

**Keywords:** Unmet need, Family planning, Adolescent girls and young women, Ethiopia

## Abstract

**Background:**

Unmet need for family planning among adolescent girls and young women (AGYW) is a common cause of the low contraceptive utilization in developing countries, including Ethiopia. To address problems associated with unmet for family planning among adolescent girls and young women nationally available evidences are essential. However, there is limited evidence regarding factors associated with the unmet need for family planning among adolescent girls and young women in Ethiopia. Hence, this study aims to assess the prevalence and associated factors of unmet need for family planning among adolescent girls and young women in Ethiopia.

**Methods:**

Our analysis was based on secondary data using the 2016 Ethiopian Demographic and Health Survey data. A total weighted sample of 1086 adolescent girls and young women was included in this study. A multi-level mixed-effect logistic regression analysis was fitted. Adjusted odds ratios with 95% confidence intervals were used to show the strength and direction of the association. Statistical significance was declared at a *p*-value less than 0.05.

**Results:**

The prevalence of unmet need for family planning was 28.3% (95% CI: 25.7, 31.0). Adolescent girls and young women age 15–19 years (aOR: 2.4, 95%CI: 1.3, 4.3), household wealth quantile; poor (aOR: 5.6, 95%CI: 2.8, 11.1) and middle (aOR: 2.9, 95%CI: 1.4, 6.0), had no media exposure (aOR: 2.1, 95%CI: 1.1, 4.1), and adolescent girls and young women from developing regions (aOR: 5.1, 95%CI: 1.1, 14.5) were significantly associated with unmet need for family planning.

**Conclusions:**

Unmet need for family planning was high among adolescent girls and young women when compared to the national average and the United Nations sphere standard of unmet need for family planning. Age, wealth quantile, media exposure, and region were significantly associated with unmet need for family planning. Hence, there is the need to implement consistently effective family planning policies among AGYW living in developing regions of Ethiopia. Moreover, Public health policies and interventions that improve the existing strategies to improve media exposure of AGYW on family planning issues and increase the wealth status of households should be designed and implemented to reduce the unmet need for family planning in Ethiopia.

## Background

Unmet need for family planning (FP) has been affliction for most adolescent girls and young women (AGYW) in developing countries [1]. World Health Organization (WHO) describes women with unmet need for FP as fecund and sexually active women who either wish to postpone the next birth (spacing) or who wish to stop childbearing (limiting) but are not using any method of contraception [2, 3].

According to the WHO, AGYW is a person between the ages of 15 and 24 years and are characterized by unique physical, psychological, social, and emotional changes that put their life at high risk [4, 5]. The majority ( 89%) of young women living in developing countries [6]. By the year 2050, it has been projected that the number of young people in Sub-Saharan Africa (SSA) will reach 605 million [7].

In SSA, most adolescent and young women are growing up in disadvantaged settings marked by high unemployment rates, rapid urbanization, often-limited educational opportunities, and rapidly changing socio-cultural norms and practices [8]. Apart from these general difficulties, they face a number of sexual and reproductive health issues, such as unintended pregnancies, unsafe abortions, and high fertility [8, 9].

Family planning (FP) is a means of improving health, reducing poverty, and empowering women [7, 10–12]. It can prevent up to one in every three maternal deaths by allowing women to delay motherhood, space births, avoid unintended pregnancies, abortions, and stop childbearing when they have reached their desired family size [10, 11, 13]. The ability of FP to reduce maternal deaths can be more realized if the poorest individuals and those with unmet needs are reached on a wide scale [1, 14, 15].

Globally, 12% of married or in-union women are estimated to have had an unmet need for FP [11].

In SSA, 25% of married women of reproductive age have an unmet need for FP [16]. Approximately one in every three births is an unintended pregnancy in Ethiopia due to an unmet need for family planning, with the majority of them being AGYW [17]. This high level of unintended pregnancy can result in serious health risks to mothers and their infants [17]. Reducing unmet needs would significantly reduce unintended pregnancy, abortion, and maternal and child mortality [18].

Ethiopia was working towards reducing unmet need for FP from 22% in 2016 to 10% by the end of 2020 [19]. FP2020 goals have focused on AGYW reproductive health through the provision of youth-friendly services, free contraceptives for adolescents, and ensuring consistent commodity supplies to youth-specific facilities [20]. The Ethiopian Demographic and Health Survey (EDHS) 2016 report has found that women have an average of 4.6 children [17]. This rate is more than the global rate of 2.4 children per woman [21]. Scholars found that unmet need for FP plays a key role in the high fertility rate [19, 22–24], and understand that unmet need for FP is predominant in AGYW in Ethiopia [25, 26]. It is worthwhile to know the prevalence and predictors of unmet need for FP in these high fertility rate groups [14, 22, 27–29].

Even though the prevalence of unmet need for family planning is predominate in AGYW worldwide, particularly in developing countries including Ethiopia [30, 31] and there are studies conducted on the prevalence of unmet need for family planning in Ethiopia [10, 24, 32–34], most of these studies were limited to reproductive-age women and focused on specific area. The current study used multilevel analysis to model the hierarchical nature of the data, which differed from the previous studies. Moreover, the current study tried to assess additional factors such as the terminated pregnancy and desired number of children.

As to our search of the literature, no study has been conducted to investigate the prevalence and related factors of unmet need for family planning among AGYW in Ethiopia based on the Ethiopia Demographic and Health Survey (DHS) data. Investigating the prevalence of unmet need for family planning and its associated factors in Ethiopia is crucial to assessing cross-national disparities. Besides, the study had adequate statistical power to detect the true effects of variables; hence, it is based on the EDHS data in Ethiopia. An important benefit of this study is that it will serve as input to program planners, who will use the results to allocate resources for improving maternal and child health. Therefore, the aim of this study is to assess unmet need for family planning and associated factors among AGYW in Ethiopia.

## Methods

### Study settings and data source

Data for this study came from the most recent EDHS, which was conducted by the Central Statistical Agency (CSA) in collaboration with other government agencies. The EDHS was a national representative sample conducted from January 18th to June 27th, 2016 [35].

**Fig. 1:**
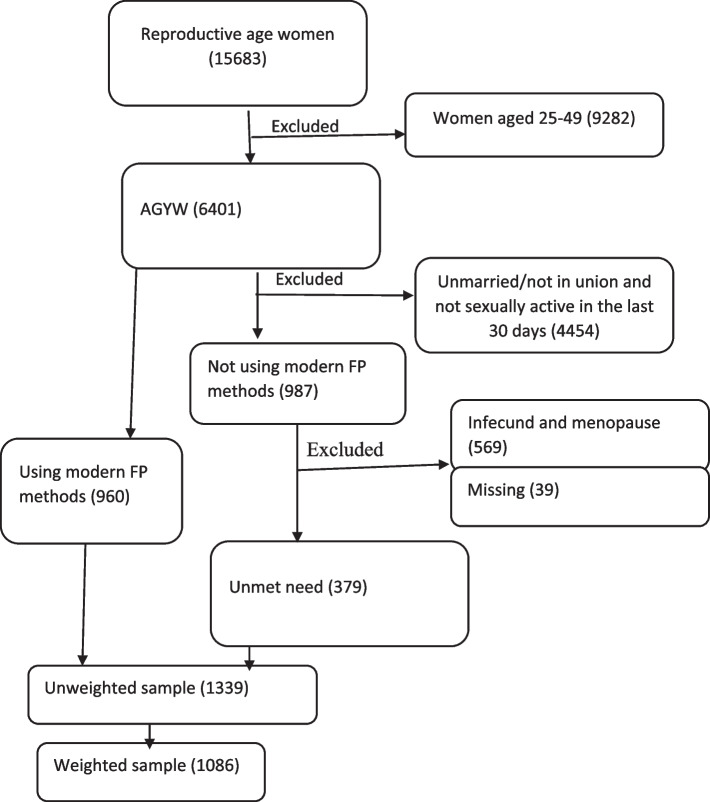
Schematic presentation for unmet need for FP among AGYW in Ethiopia

For this study, we used the women’s recode dataset and extracted the dependent and independent variables. The dataset is freely available for download at: https://dhsprogram.com/data/available-datasets.cfm. EDHS uses a two-stage stratified cluster sampling. This makes the data nationally representative [36]. Data were collected by trained data collectors using pretested structured and interviewer-administered questionnaires. The source population was AGYW during the survey in Ethiopia. Those who had never had sex, were not sexually active, and were infecund were excluded from this analysis. A weighted sample of 1086 AGYW were included in this study (Fig. 1).

### Study variables

#### Dependent variable

The dependent variable for this study was unmet need for FP, which was generated from constructed EDHS variables. It is the sum of unmet need for spacing and limiting. AGYW who are married, fecund, and/or sexually active have unmet needs if they want to delay or limit future pregnancy but do not use any form of contraception. The dependent variable was a binary variable. Those with an unmet need for spacing or limiting were coded as 1, while those using FP methods for spacing and or limiting were coded as 0 [37–39]**.**

#### Independent variables

We incorporated several individual and community level independent variables based on reviewing different related literatures**.** Age of the women (15–19, 20–24), women's education (no formal education, primary education, and secondary education and above), husband education (no formal education, primary education, and secondary and above education), religion (Orthodox, Muslim, protestant, and other), occupation of the respondents (no work, professional workers, agricultural workers, and other), occupation of the husband (no work, professional workers, agricultural workers, and other), place of delivery (home, health institutions), desired number of children (Having another, undecided, and wanted no more), terminated pregnancy (yes, no), and parity (primipara, multipara) [10, 15, 38, 40–47]**.**

The household wealth index was calculated based on consumer goods such as televisions, bicycles, and cars. The material used for the roof, floor, and toilet facilities was considered in calculating the household wealth index. The wealth index was constructed using household asset data via Principal Component Analysis (PCV) to categorize individuals into wealth quintiles (poor, middle, and rich) [48, 49]. Regarding media exposure (yes, no), we coded yes if the women read newspaper, listened radio, or watched television for at least less than once a week, and no for otherwise [50].

Of the community level factors, residence (rural, urban) and region were directly accessed from the EDHS data set. Region was categorized into two regions; developing (Afar, Somali, Benishangul, and Gambela), developed regions (Tigray, Amhara, Oromia, and Sothern Nations Nationalities and People Region, Harari, Dire Dawa, and Addis Ababa) based on their geopolitical features, indicators related to health, human development and Millennium Development Goals compared to other developed regions of Ethiopia and consistent with a previous study conducted in Ethiopia [51, 52]. However, the aggregate community level independent variables (community level poverty, community level media exposure, and community level education) were constructed by aggregating individual-level characteristics at the community (enumeration area)) level.

Community-level poverty categorized as low if the proportion of household which is from households belonging to the categories of poor was less than 50% and categorized as high if the proportion was greater than 50%. It was coded as “0” for low(communities in which < 50% of women had media exposure at least for one media), “1” for high community-level media exposure (communities in which ≥ 50% of women had at least for one media [49, 53]. Community-level education was also categorized high or low based on national media value (50% percentiles) [24].

### Statistical analysis and model building

Stata version 14 statistical software was used for data analysis. All frequency distributions were weighted using the weight command in Stata (v005/1000000) throughout the analysis to ensure that the DHS sample was a representative sample and to obtain reliable estimates and standard errors before data analysis. The first approach involved the use of percentages to describe the unmet need for FP among AGYW in Ethiopia. This was followed by the distribution of unmet need for FP across the individual and community level factors. Pearson's chi-square test of independence (X^2^) was used to assess the significance of the association between each independent variable and the unmet need for FP at a *p*-value of < 0.05. Finally, multilevel binary logistic regression analysis was done to assess the association between unmet need for FP and the individual and community-level factors. In the EDHS data, there was a hierarchical structure, which violates the independent observations and equal variance assumptions of a traditional logistic regression model. Therefore, women were nested within households, and households were nested within clusters. Within the cluster, they may have similar characteristics. Hence, multilevel binary logistic regression analysis must take into account the variability between clusters.

Intra-class correlation coefficient (ICC), Median Odds Ratio (MOR), and Proportional Change in Variance (PCV) were computed to measure the variation between clusters. Taking clusters as a random variable, the MOR is defined as the median value of the odds ratio between the area at the highest risk and the area at the lowest risk area when randomly picking out two clusters. ${{\mathrm{MOR}=e}^{0.95}}^{\sqrt{VA}}$ Whereas, the ICC reveals the variation of unmet need for FP between clusters is calculated as;$ICC=\frac{VA}{VA+3.29}*100\%$. Moreover, the PCV reveals the variation in the prevalence of unmet need for FP among AGYW explained by factors and calculated as; $PCV=\frac{Vnull-VA}{V null}*100\%$ where; Vnull = variance of the initial model, and VA = area/cluster level variance [54–56].

The fixed effects or measure of association was used to estimate the association between the likelihood of prevalence of unmet need for FP and individual and community levels independent variables. It was assessed and the strength was presented using Adjusted Odds Ratio (AOR) and 95% confidence intervals with a *p*-value of < 0.05.

$$Log \left(\frac{\pi ij}{1-\pi ij}\right)=\beta o+ \beta 1xij+ \beta 2xij+\dots uj+eij$$

where,$\pi ij$: the probability of unmet need for FP, $1-\pi ij$: the probability of met need for FP. ß0 is intercept that is the effect of unmet need for FP when the effect of all independent variables is absent. $\beta 1xij$ are individual and community level variables for the i^th^ individual in group j, respectively. The ß’s are fixed coefficients indicating a unit increase in X can cause a ß unit increase in probability unmet need for FP. The uj shows the random effect for the j^th^ clusters [54, 56, 57].

Model comparisons were done using the deviance test and log likelihood test and the model with the highest log-likelihood ratio and the lowest deviance was selected as the best-fitted model.

Moreover, multicollinearity was tested using the variance inflation factor (VIF) and we have got a VIF of less than five for each independent variable with a mean VIF of 1.55, indicating there was no significant multicollinearity between independent variables. After selecting variables for multivariable multilevel analysis, four models; the null model (without independent variables), mode I (containing only individual-level factors), mode II (Community-level factors), and model III (containing both individual and community level factors) were fitted. Variables with Adjusted Odds Ratio (aOR) with a 95% Confidence Interval (CI), and *p*-value < 0.05 in the multivariable model were considered significantly associated factors of unmet need for FP.

## Results

**Fig. 2:**
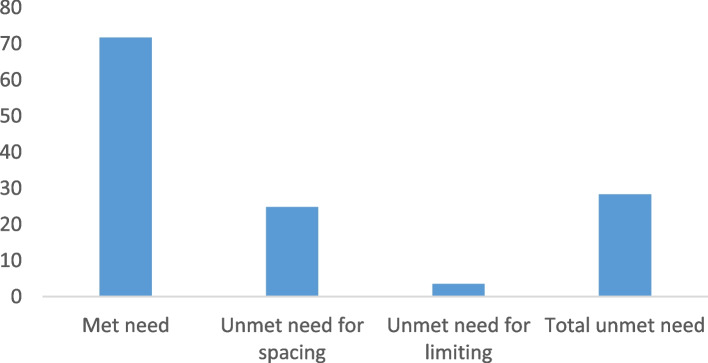
Unmet need for FP among AGYW in Ethiopia, EDHS 2016

In this study, the overall unmet need for FP among AGYW in Ethiopia was 28.3% (95% CI: 25.7, 31.0), of which 24.8% was for spacing (Fig. 2).

### Distribution of unmet need for FP across the individual and community level factors

Table 1 shows the results on the distribution of unmet need for FP across the individual and community-level factors in Ethiopia. The results indicate that the unmet need for FP was high among AGYW aged 15–19 (32.6%), those with primary level of education (39.1%), those who had no media exposure (31.1%), and AGYW in the poor wealth quintile (44.1%). A greater proportion of AGYW also had unmet need for family planning if they desired to have no more children (38.7%), those who had no terminated pregnancy (35%), and those who had multipara (38.5%). There were also variations in the proportion of unmet need for FP across the various community factors, lived in rural areas (32.7%), lived in communities with low literacy (36.6%), lived in communities with low media exposure (53.9%), livid in communities with high poverty (28.6%), and livid in developing regions (42.9%) (See Table 1).

**Table 1:** Distribution of unmet need for family planning across individual and community level factors among AGYW in Ethiopia, EDHS 2016

Variables	Weighted N	Weighted %	Unmet need for FP	X^2^ ( *p*-value)
Age				17.3 (< 0.001)
15–19	255	23.5	32.6	
20–24	831	76.5	26.9	
Educational status				54.0 (< 0.001)
Religion				
Orthodox	505	46.5	15.4	
Muslim	317	29.2	54.2	
Protestant	235	21.6	19.7	
Other	29	2.7	29.4	
No formal education	285	26.2	39.1	
Primary education	734	67.6	26.1	
Secondary and above	67	6.2	6	
Occupation of the respondents				12.7 (> 0.05)
No work	595	54.8	31.3	
Professional workers	206	19	12.1	
Agricultural workers	262	24.2	34.1	
Other	23	2	28.2	
Household wealth				144.4 (< 0.001)
Poor	408	37.6	44.1	
Middle	218	20	29.8	
Rich	246	22.7	13.6	
Media exposure				386.5 (< 0.001)
Yes	801	26.2	20.4	
No	285	73.8	31.1	
Parity				67.3 (< 0.001)
Primipara	694	63.9	22.5	
Multipara	392	36.1	38.5	
Age at first birth				26.9 (> 0.05)
< 18 years	610	56.2	31.6	
≥ 18 years	476	43.8	24	
Terminated pregnancy				21.7 (< 0.02)
Yes	49	4.5	28	
No	1037	95.5	35	
Decision maker for FP				
Mainly respondent	234	23.5	30	
Mainly husband	47	4.3	25.5	
Jointly	805	72.2	25.5	186.8 (< 0.001)
Have another	929	85.6	26.2	
Undecided	19	1.7	52.5	
Wants no more	138	12.7	38.7	
Age at first sex				43.1 (> 0.05)
< 18 years	899	82.8	31.5	
≥ 18 years	187	17.2	21.7	
Resident				52.9 (< 0.001)
Rural	902	83.1	32.7	
Urban	184	16.9	6.4	
Community level poverty				36.5 (< 0.001)
Low	102	9.4	25.5	
High	984	90.6	28.6	
Community-level media exposure				314.5 (< 0.001)
Low	507	46.7	53.9	
High	579	53.3	19.6	
Community level education				47.6 (< 0.001)
Low	506	46.6	36.6	
High	580	53.4	21	
Region				28.3 (< 0.001)
Developing	35	3.2	42.9	
Developed	1051	96.8	27.8	

### Random effects and model fitness

The intra-class correlation (ICC) in the null model indicated that 70% of the overall variability of unmet need for FP was attributed to cluster variability. The median odds ratio for unmet need for FP was 13.9 in the null model, which indicates that there was a variation in unmet need for FP between clusters. This means if we randomly select individuals from different clusters, individuals at the cluster with higher unmet need for FP had 13.9 times higher odds of unmet need for FP as compared to those individuals at cluster with lower unmet need for FP.

The proportional change in variance (PCV) also increase from 9.5% in model I to 19.24% in model III (a model with individual and community level variables), which indicates the final model (model III) best explains the variability of unmet need for FP. In addition, model fitness was checked using deviance, the model with lowest deviance (model III) was the best-fitted model (Table 2).

**Table 2:** Multi-level mixed-effect logistic regression analysis of factors associated with unmet need for FP among AGYW in Ethiopia, EDHS 2016

Variables	Null model	Model I	Model II	Model III
Age in years				
15–19		2.5 (1.4, 4.5)		**2.4 (1.3, 4.3)**
20–24		1		1
Educational status of the respondents				
No formal education		3.7 (0.8, 16.9)		2.5 (0.5, 13.2)
Primary education		2.4 (0.6, 10.1)		1.8 (0.4, 8.1)
Secondary and higher		1		1
Wealth index				
Poor		8.3 (4.3, 15.9)		**5.6 (2.8, 11.1)**
Middle		4.0 (2.0, 8.0)		**2.9 (1.4, 6.0)**
Rich		1		1
Media exposure				
No		1.5 (0.8, 2.7)		**2.1 (1.1, 4.1)**
yes		1		**1**
Desire number of children				
Having another		1		1
Undecided		3.4 (0.8, 14.7)		3.2 (0.8, 14.0)
Wants no more		1.1 (0.5, 2.0)		1.2 (0.6, 2.3)
Terminated pregnancy				
No		1		1
Yes		1.3 (0.4, 3.8)		1.2 (0.4, 3.7)
Parity				
Prime para		1		1
Multi para		(0.9, 2.8)		1.3 (0.8, 2.6)
**Community level factors**				
Community level poverty				
High			1	1
Low			1.1 (0.3, 4.6)	0.8 (0.2, 3.8)
Community media exposure				
High			1	1
Low			0.3 (0.1, 3.6)	0.5 (0.1, 5.3)
Community level education				
High			1	1
Low			1.4 (0.1, 13.9)	2.1( 0.2, 19.9)
Residency				
Urban			1	1
Rural			14.8 (3.6, 61.3)	3.6 (0.84, 15.2)
Region				
Developed			1	1
Developing			7. 04 (1.9, 25.7)	**5.12 (1.1, 14.5)**
Random effect result				
ICC	0.7	0.67	0.66	0.65
Variance	7.69	6.96	6.61	6.21
MOR	13.9	12.26	11.5	10.67
PCV	Reference	9.5	14	19.24
Model fitness				
LL	-508.4	-473	-483	-462
Deviance	1016.8	946	966	942

^*^Statistically significant at *p*-value < 0.05, Null model: adjusted for individual-level characteristics, Model 2: adjusted for community-level characteristics, Model 3: adjusted for both individual and community-level characteristics

### Factors associated with unmet need for family planning in Ethiopia

In the final model (model III) after adjusting for individual and community level factors, age of respondents, household wealth index, media exposure, and region were significantly associated with unmet need for FP among AGYW.

Accordingly, the odds of unmet need for FP among AGYW aged 15–19 years were 2.4 (aOR: 2.4, 95%CI: 1.3, 4.3) times higher than those AGYW aged 20–24 years. AGYW had no media exposure had 2.1(aOR: 2.1, 95%CI: 1.1, 4.1) times more odds to have unmet need for FP than those who had media exposure. The odds of unmet need for FP among AGYW who lived in the developing regions (AOR: 5.1, 95%CI: 1.1, 14.5) were higher than those of AGYW who lived in developed region. The odds of unmet need for FP among AGYW from households classified as poor and moderate status were higher than those of AGYW from rich households (aOR: 5.6, 95%CI: 2.8, 11.1) and (aOR: 2.9, 95%CI: 1.4, 6.0), respectively (Table 2).

## Discussion

This study was conducted to determine the prevalence of unmet need for FP and associated factors among AGYW in Ethiopia. The current study revealed that the prevalence of unmet need for FP among AGYW in Ethiopia was 28.3% (95% CI: 25.7, 31.0). Nearly one fourth of AGYW had an unmet need for spacing and 3.5% of AGYW had an unmet need for limiting. Hence, the current study suggests that FP policymakers should focus on AGYW due to the high unmet need for FP, which exposes them to unintended pregnancies and unsafe abortions. This increases the risk of maternal and child morbidity and mortality. The result of this study showed that age of respondent, household wealth index, media exposure, and region were significantly associated with unmet need for FP among AGYW in Ethiopia.

The current finding is higher than that of earlier studies conducted in Ethiopia [10, 26, 33, 58, 59], Burkina Faso (18.26%) [60], Malawi (21%) [61], Guinea (8.6%) [40], SSA (26.6%) [29], Cambodia (11.7%) [22]. This discrepancy might be attributed to the difference in the target population and study setting, and socio-demographic differences. The current study, for example, only includes AGYW, and evidence shows that most Sub-Saharan African countries, including Ethiopia, are far from adequately meeting the needs of family planning in their AGYW population and lack the knowledge, agency, or resources to make reproductive decision [42, 47]. Regarding study setting, for example, the previous studies done in Ethiopia were small-scale surveys compared to the EDHS survey, which was a national representative survey and included peripheral regions. Another possible explanation might be the socio-demographic and economic differences among study participants. For instance, a previous study done in Guinea reported that only 0.4% of the women had the poorest wealth quintile, which was lower than that of the current study (14.6%). Previous research has shown that the wealth quintile has a negative relationship with unmet FP need [10, 44, 62].

However, this study also lower than studies conducted in Nigeria 35% [45], Cameroon 46.6% [15], Ghana 35.17% [63], and Angola 51.7% [46]. This variation might be because the variation in time gap and socio-demographic characteristics, for example, the proportion of women who have poorest wealth quintile in this study was 14.6%, which was lower than that of the study conducted in Angola (36.1%). Moreover, the proportion of AGYW who have no formal education in this study was 26.2%, which was lower than that of the study conducted in Ghana (34%). In this regard, previous studies found that educated women had less likely to have unmet need for FP than uneducated ones [10, 44, 46, 64]. Therefore, having a low proportion of women who had no formal education in our study may reduce the odds of unmet need for FP.

Accordingly, in this study, it was found that the likelihood of unmet need for FP was high among AGYW aged 15–19 years compared to those aged 20–24 years. This is congruent with studies done in Ghana [63], and SSA [44]. The possible explanation might be attributed to the socio-cultural norms surrounding access to FP among adolescents in developing countries including Ethiopia [65–67]. Moreover, adolescents aged 15–19, compared to young women aged 20–24 years are less likely to be start childbearing and might have the desire to delay/limit childbearing, those who have more desire to limit or delay childbearing can experience unmet need for FP [68, 69].

The likelihood of unmet need for FP among AGYW from households with poor and middle wealth quintile were higher than those from households with rich wealth quintile. This finding is supported by studies done in Ethiopia [39], Ghana [62], and SSA [44]. The reason might be AGYW from poor and middle households cannot deal with the cost barrier associated with access to family planning as compared to those from rich households since they cannot able to overcome both the direct and indirect cost associated with family planning uptake [43, 70].

Media exposure associated unmet need for family planning among AGYW. Women who had no media exposure were more likely to have unmet need for family planning as compared to their counterparts. Where exposure to mass media had a substantial positive effect on FP use and intended future use of FP. This may be due to the reason that women who did not have media exposure might not have a batter awareness about where to access family planning services and products [71].

This study revealed that the AGYW from developing regions were more likely to have unmet need for FP than those from developed region. Evidences in Ethiopia revealed that sexual and reproductive health services are varied across regions, where many of the sexual and reproductive health services including contraceptive remains very low in developing regions [72]. For instance, according to the 2016 EDHS report, contraceptive coverage was 2% in Somali region (peripheral region) whereas 56% in Addis Ababa (metropolitan) [72]. Moreover, evidence revealed that there is a high concentration of sexual and reproductive health services delivery in developed regions in Ethiopia, which in turn can make it difficult for AGYW from developing regions to get sexual and reproductive health services and have an impact on individual life [12, 26, 73].

### Strength and limitation of the study

The strength of the current study was that it used nationally representative survey data with large sample size. We employed multilevel analysis to accommodate the hierarchical nature of the data. However, the temporal relationships between the dependent variable and independent variables could not be established due to the cross-sectional nature of the study. EDHS are based on self-reported information, which is likely to introduce social desirability bias. The other limitation of the current study is that, since we used secondary data, some important variables like family or husband opposition, knowledge about family planning, and side effects were not included in the analysis.

## Conclusion

Our study has demonstrated that unmet need for family planning among AGYW was high when compared to the national average and the United Nations sphere standard of unmet need for family planning. This can result in high rates of unintended pregnancy and unsafe abortion and an increased risk of contracting STIs, including HIV/AIDS. This raises the risk of maternal and child morbidity and mortality. Age of AGYW, wealth quantile, media exposure, and region were significantly associated with unmet need for FP. Hence, there is the need to implement consistently effective FP policies among AGYW living in developing regions of Ethiopia. Moreover, Public health policies and interventions that improve the existing strategies to improve media exposure of AGYW on FP issues and increase the wealth status of households should be designed and implemented to reduce the unmet need for FP in Ethiopia.

## Data Availability

Data for this study were sourced from Demographic and Health surveys (DHS), which are freely available online at (https://dhsprogram.com).

## Abbreviations

AOR: Adjusted Odds Ratio
AGYW: Adolescent Girls and Young Women
EAs: Enumeration Areas
EDHS: Ethiopian Demographic and Health Survey
FP: Family Planning
ICC: Intra-class Correlation Coefficient
MOR: Median Odds Ratio
PCV: Proportional Change in Variance
SD: Standard Deviation
WHO: World Health Organization
